# A new translational target for deep brain stimulation to treat depression

**DOI:** 10.1002/emmm.201302947

**Published:** 2013-07-04

**Authors:** Karl Kiening, Alexander Sartorius

**Affiliations:** 1 Division of Stereotactic NeurosurgeryDepartment of Neurosurgery, University Hospital HeidelbergHeidelberg, Germany; 2 Department of Psychiatry and PsychotherapyCentral Institute of Mental HealthMedical Faculty Mannheim, University of HeidelbergMannheim, Germany

**Keywords:** DBS, depression, lateral habenula, Learned Helplessness, treatment resistance

Depression is a common, severe psychiatric illness with an enormous individual and societal burden and limited therapeutic options. Unfortunately, between about 10 and 30% of depressed patients taking antidepressants and receiving psychotherapy are partially or totally resistant to these treatments, and even with electroconvulsive therapy, a significant number remain refractory between about 10 and 30% of depressed patients taking antidepressants and receiving psychotherapy are partially or totally resistant to these treatments, and even with electroconvulsive therapy, a significant number remain refractory. Thus, there is an urgent need for more effective treatments, especially for the most severely affected fraction of patients.

…between about 10 and 30% of depressed patients taking antidepressants and receiving psychotherapy are partially or totally resistant to these treatments, and even with electroconvulsive therapy, a significant number remain refractory.

For the treatment of neurological symptoms, such as essential tremor, dystonia and Parkinson Disease, deep brain stimulation (DBS) has become a routine treatment. Given this success, the last decade has seen its application in small case series and individual cases to treat depression. Most of these studies have described successful treatment rates of around 30–50% in patients who had previously been characterised as treatment resistant.

Such an approach in treatment resistant depression (TRD) of course depends on the selection of suitable targets in the complex neural circuits mediating this brain disease. To be able to translate preclinical findings into human research, a valid animal model and a tool for translation are required. In our search for an appropriate target, we have used the congenital Learned Helpless (cLH) model, which uniquely models TRD. *Inter alia*, our established genetic strain of Sprague-Dawley rats demonstrates not only specific helpless behaviour, but also anhedonia (Enkel et al, 2010), depressive-like ‘pessimistic’ cognitive bias (Richter et al, 2012), treatment resistance (even to electroconvulsive shock therapy), as well as histological and biochemical changes, such as alterations in hippocampal glutamatergic and GABAergic systems.

The lateral habenula (LHb; e.g. Morris et al, 1999) is hyperactivated in patients with a unipolar depressive disorder undergoing tryptophan depletion (tryptophan is an essential amino acid necessary for serotonin synthesis). Basic research into the LHb indicates that it is linked to stress and pain perception, the reward system, diurnal regulation and sexual functioning (Hikosaka, 2010). It is also known to directly affect all three monoaminergic brain systems, including the serotonergic one—directly via the dorsal raphe nuclei. Stereotaxic pharmacological (Winter et al, 2011) and stereotaxic electric (Li et al, 2011) inhibition of the LHb have been found to lead to the improvement of depressive-like behaviour in translation studies using a model of depression with the Learned Helplessness paradigm.

**Figure 1:**
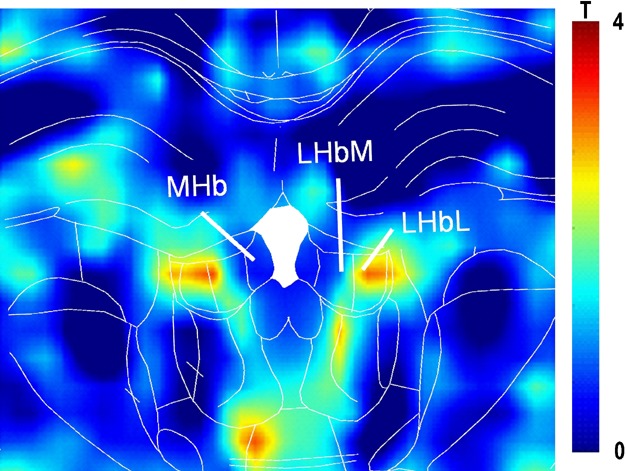
An example of ‘genetic imaging’.This map shows differences in regional cerebral blood volume (rCBV; a voxel-by-voxel based statistic was performed and is shown as a T-map) of two groups of genetically different animals (cLH: *n* = 8 and cNLH: *n* = 7). Significant alterations of rCBV within the lateral part of the LHb (acquired *in vivo* with a 9.4 T magnetic resonance scanner) are shown. LHbM—lateral habenula, medial part; LHbL—lateral habenula, lateral part; MHB—medial habenula.

To further elucidate brain function as an endophenotype, we used functional high-field magnetic resonance imaging at 9.4 T as a translational tool to look for disease specific alterations, which again led to the observation of functional alterations of the LHb. Fig 1 demonstrates a significant difference in habenular cerebral blood volume between animals genetically prone to show depressive-like behaviour (cLH) versus animals that are not (cNLH). Since animals do not behave during measurements (they have to be sufficiently, albeit lightly, anaesthetised within the scanner), imaging differences can be attributed to genetically induced alterations of brain function.

As a result of our work and other growing evidence that the overactivation of the LHb is a necessary condition for the state of depression in patients or depressive-like behaviour in animals (Sartorius et al, 2010)—and assuming that high frequency DBS of white matter bundles would lead to a functional inhibition—we decided to use bilateral DBS of the LHb in two patients with severe therapy-refractory depression for whom years of treatment, including electroconvulsive therapy, had failed to have a sustained impact. This is the first such use of this therapy. More precisely, we put electrode tips into the stria medullaris, which is the main limbic afferent white matter bundle of the LHb. This 1:1 translational step resulted from the excellent validity of our cLH animal model, but also from other animal and human findings to date, all of which suggest that the LHb has to be downregulated to cure depression.

DBS of this small midline structure—or rather, its major afferent bundle adjacent to the 3rd ventricle—presents predictable, but solvable challenges to the neurosurgeon (Schneider et al, 2013). Preventing intracranial bleeding while introducing the electrode into the brain is the most important issue that warrants precise anatomical planning. The pacer itself is anatomically implanted, much like a cardiac pacemaker.

The first patient remitted from all depressive symptoms with DBS, but showed two full relapses when the pacer malfunctioned (the patient was not aware of the malfunction; Sartorius et al, 2010). The second patient has improved more than 50% on a depressive symptom scale so far, but suffered from a comparable relapse during an OFF period due to a pacer exchange (because of a pacemaker infection).

Strikingly, all of the (DBS-OFF) relapses were rapid in their onset—about 1 week—and had slow remission times—more than 8–12 weeks after DBS-ON. This sort of pattern has also been seen in patients treated with DBS of the subgenual cingulate. Interestingly, the subgenual cingulate, as well as the medial forebrain bundle, is part of the midline neurocircuitry that potentially affects the LHb in the same (suppressive) direction. As such, we believe that the uneven time course observed in the patients in our DBS study—very fast relapses followed by slow remissions—is likely linked to alterations of neuroplasticity and perturbations of certain neurocircuits that are pertinent to modern theories of depression.

Notably, in the first patient, DBS led to an increase in the brain-derived nerve growth factor (BDNF) serum level. BDNF is a member of the ‘neurotrophin’ family of growth factors, and various treatments for depression—such as antidepressants and electroconvulsive therapy, as well as sleep deprivation—have been shown to increase the expression of BDNF in the brain. Interestingly, the direct injection of BDNF into rat hippocampi has been shown to improve depressive-like behaviour.

Changes in the levels of neurotrophins such as BDNF are the basis of the neurotrophin hypothesis of depression, which posits that stress and depression lead to impaired neuroplasticity, and that antidepressive treatments increase neuroplasticity. This corroborates the idea that changes in the rate of synaptic and neuronal formation might underlie depressive illnesses (Henn et al, 2004).

Recently, the significant influence of epigenetics in numerous cellular processes and psychiatric disorders has been recognised. Histone acetylation and methylation show differences in various brain regions of animal models of anxiety and depressive-like behaviour. Nevertheless, research regarding ‘imaging epigenetics’ has only just begun—after the groundbreaking success of human imaging genetics (e.g. Esslinger et al, 2009). On top of this, imaging seems to be a promising translational tool to directly and non-invasively endophenotype the brain function of patients and animals, as well the changes that result from genetic, epigenetic, environmental or treatment interventions.

In conclusion, our use of a valid genetic animal model (cLH) of TRD, combined with a highly translational approach has allowed us to successfully introduce DBS of the LHb in patients suffering from TRD, demonstrating for the first time the feasibility of this target and approach in humans.

…our use of a valid genetic animal model (cLH) of TRD, combined with a highly translational approach has allowed us to successfully introduce DBS of the LHb in patients suffering from TRD
